# Digitalis—Is It a Cardiac Sedative, or Cardiac Stimulant?

**Published:** 1874-12

**Authors:** T. Curtis Smith

**Affiliations:** Middleport, Ohio


*Digitalis—Is it a Cardiac Sedative, or Cardiac Stimulant?* By T. Curtis Smith, M.D., of Middleport, Ohio.

Digitalis has been prominently before the profession since 1775, at which time it was introduced by Dr. Withering, in a monograph treating of its value in dropsy. It has held a place in the British Pharmacy since 1721. It has, however, been believed to be by most early writers a cardiac sedative, and is still so considered by some writers, and many, or most of the profession, at the present day. To give a comparative statement of authorities as to its physiological effects and therapeutic value and applications, are the chief objects of this paper. We shall also add some of our own experience with the agent, in the treatment of diseases to which it seemed applicable.

Dr. Eberle, (*Therapeutics*, 1842) says: "I am entirely persuaded that its operation is immediately sedative; for it is certain that its stimulant effects, if it has any at all, are extremely feeble, and by no means proportionate to its ultimate sedative influence." "The fact of the sedative influence of digitalis (Ferriar) is so decisive that I do not hesitate to employ this term, notwithstanding the jargon with which the public has of late years been abused on the subject of sedatives." In the time when Ferriar wrote, there was great discord as to the properties of digitalis, as it was variously claimed to be a diuretic, a stimulant, or a sedative, while some claimed that it had "no properties at all."

Drake, Fowler, Beddoes, Mossman, Stefford, Magennis (loc. cit.), and others, extolled it as a remedy *par excellence* in pulmonary phthisis. This we now know to be untenable. All these writers believed in its cardiac sedative power.

Drs. Hill and Cox recommended it in mania, on account of its sedative effects.

Dr. Baildon (*Edinburg Med. Jour.*, vol. iii.) seems to have been the first to notice the variation of the pulse with different postures of the body. Thus, while standing, the pulse run at 110; while sitting, 70; while lying down, at 40. This effect was in his own person, but also observed to be the case with other individuals. This difference, it was thought by him and others, accounted for the difference of opinion in different observers and experimenters, and especially for that of Saunders, who looked upon the agent as a cardiac stimulant, prior to 1830.

Dr. Geo. B. Wood (*Therapeutics*) believes the agent to be a local excitant, and that any temporary increase of the pulse is caused by sympathetic action from the local effects of the drug on the stomach, which is sometimes inflamed by too free use of it; but that its effect on the general system is that of a sedative to the nervous centres and to the circulation, the latter being under the control of the former. "The evidence (loc. cit.) of its primary influence upon the nervous centres, and through them on the heart, is afforded by the fact already stated, that if the par vagum be divided on both sides, it (digitalis) ceases to reduce the heart's action, and in moderate doses produces no effect at all."

He further states that with which nearly every writer agrees, viz: That it diminishes the frequency of the pulse. But contrary to the careful observation of others, who have conducted thorough physiological experiments with it, he denies that it increases the volume of the pulse or its tension. "On the contrary," he states, "the resistance under the fingers is generally, if not invariably, diminished in the normal state of the system; and if the influence of the medicine is increased, it may become extremely feeble."

Wood, in referring to the experiments of Traube, of Berlin, with digitalis, in which the pneumogastrics on both sides were divided, and in consequence of which the drug seemed to lose its effects over cardiac action, concludes from this that the influence it exerts is wholly through these nerves, seeming to forget what influence the cardiac ganglia of the sympathetic system has over the movements of the heart. Of this latter point we shall have more to say further on. The remarkable depressing effect which digitalis has over the sexual organs, Wood also refers to its (*London Medical Times and Gazette*, April, 1855,) sedative influence over the organic nerves. "The appearances after death (from digitalis, loc. cit.) are marks of inflammation in the stomach and bowels, dark and coagulable blood, and a *loss of contractibility in the heart.*" (Italics mine.)

This is directly contrary to the carefully-performed experiments of J. Milner Fothergill (Monograph on Digitalis, its Mode of Action and Uses—Hasting's prize essay for 1870), at least as far as the left side of the heart was concerned; for in animals poisoned by it, the left side of the heart was always found empty, and in a state of tonic contraction.

We shall have occasion to refer to these experiments further on.

Waring also adds his view of the sedative action of digitalis, also referring, as do all others, to its cumulative action in some cases, and to its diuretic powers.

Dr. Garrod thinks it a much better diuretic where a deficiency of urinary secretion depends on cardiac disease. This is probably true, as we shall soon try to explain. Waring (Therapeutics, 2nd Lond. Ed., 1866,) also states that if its sedative or diuretic effects are observed, the patient is free from danger of its cumulative power, in which opinion Dr. Munk (Guy's Hospital Reports, 1844,) concurs.

Notwithstanding the sedative action so strongly set forth by Waring (loc. cit.), he states as his fifth proposition that "it is chiefly applicable to diseases of an asthenic character, and in persons of shattered and debilitated constitutions." This to me seems strangely inconsistent; for patients who are "asthenic," and have shattered and debilitated constitutions, are not those to whom I would feel like administering powerful sedatives, but rather the reverse, unless for some special systemic conditions.

To add to this strange inconsistency, we will quote his seventh proposition, viz.: "In old persons, it is necessary carefully to watch the action of digitalis. It has been observed by Schonlein to produce, in some instances, not only an *alarming weakness, but a positive wasting and marasmus*, probably by acting on the nervous system and organs of digestion." Still he has just said it is best adapted to patients that are asthenic, and whose constitutions were in a shattered and debilitated condition. Certainly to make out both of the above propositions must require a mind able to twist and worm around direct contradictions that surpasses that of most men. We think that we shall be able to prove that it is best adapted to those cases where the central organ of circulation is affected with a disease that has weakened its action, and especially where that disease affects mostly the left side of the heart, and shall do so partly from those very authorities that hold it to be such a powerful cardiac sedative, either directly or through the nervous centres. For instance, Dr. Withering (1775), as quoted by Waring, observes: "That it seldom succeeds (in dropsy) in persons of great natural strength or plethoric habit, or in those with a tight and cordy pulse. If the belly in ascites be tense, hard, and circumscribed, or the limbs in anasarca solid and resisting, we have but little hope. On the contrary, *if the pulse is feeble and intermitting* [italics mine], the countenance pale, the lips vivid, the skin cold, the swollen belly soft and fluctuating, the anasarcous limbs pitting under pressure of the finger, we may expect the diuretic effects to follow in a kindly manner." To which Waring adds in comment: "Experience has fully proved the general justness of Withering's remarks." More of these citations hereafter.

Let us now turn to the investigations of J. Milner Fothergill, who, I think, has physiologically demonstrated, and by actual practical experience with the afflicted, proven that it is not a cardiac sedative, but a cardiac tonic of no mean consideration, and that its value in heart diseases, where that organ is weak and debilitated, or doing overwork from some structural change or circulatory interference, is inestimable. The quotations taken from this author will be found in a very able prize essay written by him, viz.: Hasting's Prize Essay for 1870:

"Snails, when touched with the tincture or strong infusion, took a contractile spasm, threw off a coating of mucus, and passed on apparently unaffected." Earth-worms and wasps were not affected by it.

Fishes—"minnows"—that were poisoned with digitalis, in dying were "drawn to one side." "After death, the ventricle was found firmly contracted and glistening like a speck of gristle; and, on being examined by a microscope, no cavity was visible. The auricle was distended, and vainly tried to drive any blood into the tightly contracted ventricle, the blood merely regurgitating into the venous sinus behind, and then flowing back again from the venous distension, relieving itself on the auricle in diastole." When the venous sinus was pricked, so as to permit the escape of the blood contained in the auricle, it also contracted firmly, so that under the microscope no cavity was discernible.

Two sparrows were poisoned by the drug, and "on opening them immediately after death, the *left* ventricle in each was found firmly contracted; the lungs so congested as almost to be hepatized; the *right* ventricle full of blood. It was evident that the condition of the lungs and right ventricle was due to inability to drive the blood into the contracted left ventricle. The gorged condition of the lungs accounted for the gasping respiration observed."

Experiments on mammals by Hanfield Jones and Fuller, proved that a similar condition of the heart obtained after death from digitalis (loc. cit.)

In Fothergill's experiments on frogs, the left ventricle was uniformly found to be firmly contracted, with the left auricle and right ventricle distended, and lungs greatly congested, and his experiments confirmed those of many other able physiologists, as Dybkowsky, Pellikan, Hilton Fagge and Stevenson, of England.

All these experiments being performed with the heart in plain view, it was noticed that the number of contractions were first increased and then became slower, the dilatation or diastole of the left ventricle becoming less and less perfect, until it ceased in a firm and permanent contraction—systole. Other drugs, as belladonna, caffeine, strychnine and aconite, were experimented with. Under belladonna, the contraction of the left ventricle was quite but less distinct than under digitalis; caffeine still less so; while strychnine produced no perceptible effect on the heart; while with aconite the heart in death was arrested in diastole. This latter fact shows plainly the directly opposite effects on the heart of digitalis and aconite, and leads us unavoidably to inquire whether the one will or will not antidote the other in cases of poisonings.

On this point, Fothergill's experiments go to demonstrate (loc. cit., p. 6,) that while digitalis seems completely to counteract poisoning by aconite, that aconite has not such complete control over poisoning by digitalis, though it "certainly had an influence," but did not ultimately prevent the permanent contraction of the left ventricle, which causes the fatal result under poisonous doses of digitalis. I can scarcely do better than to give his own words with reference to the counteracting influence of digitalis over aconite. The reader will remember that these experiments were performed with the heart of the frog exposed to plain view. He says: "On the contrary, the administration of digitalis was followed by the most marked results when aconite had been given, and the ventricle had become gradually more and more distended, and its contractions more and more imperfect, each contraction merely expelling a small quantity of blood off the top of the distended ventricle, the contractions becoming slower and slower, and less and less perfect, until a condition of advanced dilatation had been artificially produced; and even when the heart seemed to have given up all action, and remained in diastole, distended with blood and inert, . . . . the first effect (of digitalis) was to produce an imperfect contraction at long invervals; then the intervals became shorter and the contractions more complete, some irregularity, both as to time and amount of contraction, being observed. Slowly and gradually, however, the distended ventricle recovered itself under the action of digitalis, the contractions being more rhythmical and perfect, and the distension less and less pronounced, until a return to normal contraction and distension was brought about."

Here, then, we have an antidote for aconite poisoning, which will render us valuable aid in accidents of this character. But care must be taken not to be too lavish in the use of the antidote, else we may poison the patient with it and cause his death with a contracted left ventricle, instead of permitting such an unhappy result by dilatation, under the poisonous effects of aconite. These experiments with digitalis were also confirmed by Brunton and Gamgee, both eminent observers. They also detected "a temporary murmur" (loc. cit., p. 7), "which was probably caused by imperfect closure of the mitral valve, through the imperfect action of the musculi papellares." The normal action of the heart is controlled by the pneumogastric nerve (from the organic system), and the cardiac ganglia of the sympathetic system. These, by nerve filaments, communicate with each other. The function of the pneumogastric, with reference to that portion which is distributed to the heart, seems to be to prevent too frequent or too rapid action and too great distension, and only admits the systolic action when the ventricle becomes sufficiently distended by blood being poured into it sufficiently to stimulate the heart to contraction. The cardiac ganglia of the sympathetic, on the other hand, tend to increase the number of contractions. When the pneumogastrics are cut on both sides, the heart beats more rapidly than under the normal condition of these nerves. The heart, then, seems to be kept in normal action between these two balancing nerve powers. Digitalis evidently produces its influence over the heart secondarily, or through its action on the nerve centres controlling its action.

When the system is under the influence of digitalis, and the pneumogastrics are severed, the number of contractions are increased, but not to the same extent as when not influenced by the drug. It does not, therefore, act wholly and only on the vagus, as Traube and others have supposed. "There is no apparent connection between the giving of digitalis and section of the vagi, except an increase in arterial tension," (loc. cit.) Traube had supposed that the effect of the drug was produced by partially or completely paralyzing the vagi. But Hanfield Jones, Fuller, Neimeyer, Dybkowsky and Pellikan believe its effects to be exerted through stimulation of the cardiac ganglia, and that this stimulant effect manifests itself through increased muscular action in the heart, which is under their control. With this view Fothergill also sides, and says: "The action of the stimulated cardiac ganglia (under digitalis) is too much for the action of the vagus." Again, "The results, too, of the administration of digitalis after aconite poisoning had been established, would necessitate the theory of aconite acting as a stimulus to the fibres of the pneumogastric, if Traube's theory of the action of digitalis were true—a conclusion which is not warranted by what we know of aconite."

The action of this agent on the capillaries and arterioles is to produce an increase of their calibre, which effect still further refutes Traube's theory, as these vessels do not contract under an agent which produces a depressing effect, but dilate under such depression. This still further, also, proves that aconite depresses by nerve-sedative power, from the fact that dilatation of these vessels is very noticeable under its influence. This effect of aconite is still further confirmed by Dr. Brunton, London, in a paper recently read before the Medical Society of London, (*Medical and Surgical Reporter*, Aug. 8, 1874,) in which he commends it as our best antiphlogistic, short of venesection, and where control of the circulation is demanded without blood-letting being indicated, as is often the case in inflammatory diseases.

So much, then, for the physiological effects of digitalis upon the animal economy. The question next arises: Will these effects be proven by clinical experience? And to this we will next turn our attention; for this proof, if we can attain it, will confirm us in the views we have set forth, and place the drug in an entirely different light from that in which it has stood before the medical profession for the last century or more, and will give it a new and increased value as a therapeutic agent, with which we may be able more successfully to combat a class of diseases that have been, and still are, the opprobrium of the profession.

As we stated some pages back, we will prove, to some extent, by those who look upon this agent as a cardiac and nerve sedative, that it is not a sedative, but a cardiac stimulant and tonic, and that its value as a diuretic is based really upon the toning influence it has over the circulation, rather than that it has any specific effect as such, and we think we will, too, decidedly establish these facts by later authorities.

The belief that digitalis was a cardiac sedative, grew out of a mistaken pathology. In former times, it was believed that irregular actions and palpitations were due to or caused by "over-action of the heart," whereas we now know that they arise from a condition directly the reverse of that, and grow really out of cardiac deficiency, and that we are not now prepared to admit too great action of this organ resulting from disease of its own structure. What, you may ask, is the strong pulse of the hypertrophied heart but over-action? It is but a compensating growth to meet or overcome some obstacle, or to compensate for loss of power from dilatation of the ventricle. Fothergill says: "We can no more imagine a heart undergoing spontaneous, uncalled-for hypertrophy, than we can fancy a similar action going on in the bladder or bowel," or in any of the muscles of the body. Irregularity and palpitation are only evidences that the organ is being unduly taxed, and that whether hypertrophy exists or not—for if the two co-exist, it is evidence that the hypertrophy has not become great enough to meet the full demands of tension made on the heart by the amount of blood that passes through it. In these conditions, digitalis gives a slower and more regular action by giving tone to the weakened organ, and causing an approximation to the natural strength.

In this state of the heart, the same authority says: "Under digitalis, the pulse becomes steadier, firmer, and less compressible; the excited stroke of palpitation is steadied into the normal, quiet, effective contraction; the system is relieved; dyspnœa, the external evidence of pulmonary congestion, is abated; the *deficient secretion of urine*, (italics mine) which tells us that the pressure on the glomeruli of the malpighian bodies is lessened, is improved, and free secretion takes place; dropsy is thus often relieved."

In this we see a plain hint of the manner by which it brings about its diuretic power. It is observed that he also states that the pulse is firmer and less compressible under the influence of digitalis, which statement is directly opposite to that of Wood, (*Therapeutics*) viz.: that he had never noticed that it increased the force or tension of the pulse, which latter, also, accords with nearly all the statements of the old and some of the new writers on this subject. Fothergill adds to the above quotation the following: "The general condition of cyanosis is abated; there is evidence of a better circulation throughout the system generally. Frequently the gradually widening circle of troubles which are involving the patient's existence, diminishes after an improvement is inaugurated in the circulation." All this he attributes to "the administration of a drug whose action is unquestionably to produce better, more complete ventricular action; and in that, and that only, I believe the magic lies," (loc. cit.)

In those cases, then, in which the heart is weak, attended by an ill feeling in that region, with anxiety, breathing more or less difficult and irregular, and readily increased by physical exertion or mental excitement, accompanied with palpitation or irregularity of the pulse, skin more or less cyanosed, we may expect beyond a reasonable doubt that digitalis will prove very greatly beneficial.

Drs. Munk, Hope, R. B. Todd, Boillard, Wood, and a host of old writers, speak in warning terms of its use in the above class of cases; while Hanfield Jones, Fuller, Sutton, Gull, Wilks, Sidney Ringer, Fothergill, Gascoigne (*Brit. Med. Jour.*, Aug., 1868), Edward Mackey (*Brit. Med. Jour.*, May, 1868), and many others of equal authority, speak of it in high terms as a heart tonic or stimulant; and commend it for all diseases affecting that organ, where it has its origin in debility of its muscular fibre.

Dr. Darwell, who believed in its sedative action, nevertheless recommends it, with iron, in dropsy, following scarlet and other debilitating fevers. Why thus commend it if it is a cardiac sedative in this class of cases?—for certainly the heart partakes very largely of the general debility of the system. Dr. Holland, of the same belief, strongly commends it in similar conditions. Wood, also, speaks in favorable terms of it in dilatation of the heart, associated with anemia (certainly a condition in which the heart is much weaker than normal), where there is excessive action, and in dropsies depending on deficient cardiac power, he commends it as a diuretic; apparently overlooking the fact that its diuretic influence is attāined through the influence which digitalis has in toning the circulation, or he perhaps taking the opposite view. In any light, the above clinical facts given by these and many other men, do not correspond in result with their theory of the action of the drug, *i. e.*, as a sedative. If it is a sedative as powerful as is claimed by some, why did it not retain its reputation in inflammatory diseases, which we know it has completely lost, while its opposite, aconite, is fast gaining ground as a valuable and powerful means of combatting inflammatory action, as in acute pneumonia, pericarditis, etc., as testified to by Brunton, and others, after long experience with it at the bed-side?

Dr. E. Mackey (Prof. Materia Medica Queen's College), in reply to the question, May we expect digitalis to strengthen and make regular a feebly-beating heart, or must we dread its depressing pulsation to the verge of extinction?—says: "We may expect the former in a large number of cases."—*Compend. Med. Science* for July, 1868.

Dr. Gull clinically satisfied himself clearly "that digitalis was capable of diminishing the frequency of the heart's beat, and increasing the power of the heart's impulse, and is especially useful in cases of disease of the left side, in which the action is very rapid and feeble," (loc. cit.)

Dr. Wilks remarks of a case of dilated, enfeebled heart: "This was just one of those cases where digitalis might be expected to do good," (loc. cit.)

Dr. Fuller states that "the cases of heart disease most benefited by digitalis have been those in which the heart has been weak and dilated, pulse feeble and irregular."

Professor Warren Stone says: "Digitalis is a distinct and strong stimulant to the muscles of the heart."—*New Orleans Journal of Medicine.*

Dr. Gascoigne speaks of it as being efficient by its "tonic action on the heart."—*British Medical Journal*, August, 1868.

Dr. Wiltshire speaks of it as an agent that increases the force of ventricular contraction, especially the left, and as giving force and volume to the pulse, while it diminishes the frequency.—*Compend. Med. Science.*

Dr. B. D. Taylor, of Bellevue, New York, (*N. Y. Med. Jour.*, November, 1870) says: "The tonic action of the drug (digitalis) is well known, but the present case shows its effect in restoring power and regularity almost instantaneously to a feeble heart."

Dr. Bohm, from repeated experiments, concludes that it strengthens the contractions of the heart.

M. Gourvat concludes that the involuntary muscular tissue of the heart is strengthened by it. The beats of the heart are "rendered stronger, slower and more regular by moderate doses."—*Med. and Surg. Reporter*, 1872.

Dr. Pepper says: "In cases that are suitable, the free, fearless and continued use of this drug will relieve the most alarming symptoms of failing circulation, prolong life sometimes for years, and afford incalculable comfort."—*Med. and Surg. Reporter*, vol. xxix., p. 400.

To the above, quotations from Grimshaw, Little, Ainstie, and others, could be given to add weight, but we presume enough testimony has been given to satisfy most readers on this point. Dr. Grimshaw, considering it one of our best heart tonics, as did also Dr. Little, both used it in low typhus, strictly with a view to this effect, and with happy results. The former used the infusion, so that its effects cannot in his cases be referred to the alcohol contained, as in the tincture.

Others in this and other countries have used it with more than ordinary success in delirium tremens, and in large doses (oz. ss. to oz. j). In this class of cases not only the voluntary, but the involuntary muscles have always suffered from long over-stimulation, and are, therefore, weak; and most of those who have thus used it, have done so with a direct view to its tonic and stimulant effects on the heart. Dr. Jones, of Jersey, was, I believe, the first to bring this practice to the notice of the profession.

Numerous accounts of its value in this disease will be found in the *Compend. of Med. Science* and *American Jour. Med. Sciences*, to which the reader is respectfully referred.

My own experience is about as follows: For some years of the early part of my practice, I had been so firmly impressed with its dangerous cumulative powers, that I used it with so much caution that no perceptible effect was observed from it. But while I know that it is a powerful medicine, I have long since learned that these properties have been too greatly exaggerated, and that with care, it is as safe as any of our powerful medicines. I have also been led, from clinical observations, first induced by statements from good authority, from the opinion that it was a cardiac sedative to the direct reverse, viz.: That it is one of our best cardiac stimulants and tonics. Clinically, I have observed that those cases of palpitation, irregularity of pulse and dropsy, anasarca, dyspnœa, etc., which grow out of cardiac weakness, were formerly of great annoyance to me, and I often failed to give any more than very temporary relief. But now such cases under digitalis are generally relieved with as much certainty as an ordinary intermittent fever. It is sometimes almost wonderful to see the change which this agent effects by careful and persevering use.

In one case of dilatation of the left ventricle, in a man somewhat advanced in years, there was undoubted evidence of failure of the wall of the ventricle; the pulse became weak and intermittent; at every few beats the skin became gradually more livid; anasarca made its appearance; the respiration was also greatly interfered with, etc. He had been kept on tonics, with good hygienic regulations, and good diet, for months, but grew steadily worse, and the pulsations more irregular and feeble. I placed him on free doses of the infusion of digitalis, and after getting the heart reasonably under its influence, the dose was diminished to a half drachm, and continued twice a day, which quantity he continued to take for eighteen months. Soon after its commencement an evident improvement was observable. This continued gradually in his favor, till at the end of three months he was quite comfortable, but it was continued at least six months beyond the last vestige of any troublesome heart symptoms. At this time, now four years ago, a stronger or healthier old gentleman can hardly be found.

Numerous cases of feeble heart action could be here related, and of dropsies, pulmonary congestions, etc., growing out of feeble heart, which have been relieved by digitalis, but I presume it is needless here to prolong this paper in that manner.

We will next endeavor to point out that class of cases in which it is indicated, and its contraindications, and for the present leave this subject, feeling satisfied that we have endeavored to do our duty in demonstrating that *digitalis is a decided heart tonic.*

*Indications.*—In nearly all cases of feeble pulse from feeble heart.

In dilatation, with or without compensating hypertrophy.

In valvulur insufficiency.

In mitral obstruction.

In mitral regurgitation.

In aortic obstruction.

In aortic regurgitation *doubtfully.*

In degeneration of the cardiac walls it is indicated, but must be commenced with small doses, gradually increased, and closely watched.

In neurosal angina pectoris, not connected with serious structural degeneration.

In cardiac asthma or false angina pectoris it is valuable.

In cardiac asthma following or resulting from severe acute disease.

In shock it is valuable.

In palpitations not purely functional, it is not admissible, or is contraindicated.

In tricuspid regurgitation.

In tricuspid obstruction.

In greatly degenerated walls, or in great degeneration of the arterial coats.

In hypertrophy from plethora, with little observable dilatation, its use is questionable.

In most if not all functional disorders its value is questionable, except in cases of quite feeble pulse.

Its use as a diuretic is indicated where there is loss of tone in the circulation as a cause of the scant secretion of urine.

The modes of action as produced by digitalis are given by Fothergill as follows :

"The series of altered actions consequent upon increased contraction run in the following order or sequences; and it may be desirable for the sake of lucidity to arrange in a series of propositions, each depending on the one before, like a logical syllogism. The effects of increased contraction are, then :

"1. Increased arterial distension and tension, which give rise to the systemic symptoms, and further cause

"2. Increased arterial recoil. This is the propelling power for the coronary arteries ; and thus arterial recoil means—

"3. Increased or improved coronary circulation; and that, in turn, produces

"4. Increased nutrition of the heart, which results in

"5. Compensatory hypertrophy. In connection with these, we have also to consider—

"6. Atheroma and fatty degeneration of the heart fibres."

These propositions by their study show that digitalis follows the line which nature indicates by her attempts to restore or regulate the circulation of the system in heart diseases. So long as these indications are closely followed in its use, they will prove of the greatest value to the subjects of cardiac disease.—*Southern Medical Record.*

